# Exendin-4-Conjugated Manganese Magnetism-Engineered Iron Oxide Nanoparticles as a Potential Magnetic Resonance Imaging Contrast Agent for Tracking Transplanted β-Cells

**DOI:** 10.3390/nano11113145

**Published:** 2021-11-21

**Authors:** Jyuhn-Huarng Juang, Chia-Rui Shen, Jiun-Jie Wang, Shu-Ting Wu, Sung-Han Lin, Chen-Yi Chen, Chen-Wei Kao, Chen-Ling Chen, Zei-Tsan Tsai, Yun-Ming Wang

**Affiliations:** 1 Division of Endocrinology and Metabolism, Department of Internal Medicine and Center for Tissue Engineering, Chang Gung Memorial Hospital, Taoyuan 33305, Taiwan; Je3474@gmail.com (C.-Y.C.); lian8807111@gmail.com (C.-W.K.); jenny74513@gmail.com (C.-L.C.); 2 Department of Medicine, College of Medicine, Chang Gung University, Taoyuan 33302, Taiwan; 3 Department of Medical Biotechnology and Laboratory Science, College of Medicine, Chang Gung University, Taoyuan 33302, Taiwan; crshen@mail.cgu.edu.tw (C.-R.S.); proteinwhite@livemail.tw (S.-T.W.); 4 Department of Ophthalmology, Chang Gung Memorial Hospital, Taoyuan 33305, Taiwan; 5 Department of Medical Imaging and Radiological Sciences, College of Medicine, Chang Gung University, Taoyuan 33302, Taiwan; jiunjie.wang@gmail.com (J.-J.W.); image.lin@gmail.com (S.-H.L.); 6 Department of Diagnostic Radiology, Chang Gung Memorial Hospital, Keelung 20401, Taiwan; 7 Molecular Imaging Center, Chang Gung Memorial Hospital, Taoyuan 33302, Taiwan; zeitsan@ms9.hinet.net; 8 Department of Biological Science and Technology, Institute of Molecular Medicine and Bioengineering, Center for Intelligent Drug Systems and Smart Bio-devices (IDS2B), National Yang Ming Chiao Tung University, Hsinchu 300, Taiwan

**Keywords:** islet transplantation, manganese magnetism-engineered iron oxide nanoparticles, exendin-4, magnetic resonance images

## Abstract

To specifically detect and trace transplanted islet β-cells by magnetic resonance imaging (MRI), we conjugated manganese magnetism-engineered iron oxide nanoparticles (MnMEIO NPs) with exendin-4 (Ex4) which specifically binds glucagon-like peptide-1 receptors on the surface of β-cells. The size distribution of MnMEIO and MnMEIO-Ex4 NPs were 67.8 ± 1.3 and 70.2 ± 2.3 nm and zeta potential 33.3 ± 0.5 and 0.6 ± 0.1 mV, respectively. MnMEIO and MnMEIO-Ex4 NPs with iron content ≤ 40 μg/mL did not affect MIN6 β-cell viability and insulin secretion. Positive iron staining was found in MIN6 β-cells loaded with MnMEIO-Ex4 NPs but not in those with MnMEIO NPs. A transmission electron microscope confirmed MnMEIO-Ex4 NPs were distributed in the cytoplasm of MIN6. In vitro MR images revealed a loss of signal intensity in MIN6 β-cells labeled with MnMEIO-Ex4 NPs but not with MnMEIO NPs. After transplantation of islets labeled with MnMEIO-Ex4, the graft under kidney capsule could be visualized on MRI as persistent hypointense areas up to 17 weeks. Moreover, histology of the islet graft showed positive staining for insulin, glucagon and iron. Our results indicate MnMEIO-Ex4 NPs are safe and effective for the detection and long-term monitoring of transplanted β-cells by MRI.

## 1. Introduction

People with type 1 diabetes are characterized by loss of β-cells which cause an absolute insulin deficiency and such individuals eventually become dependent on insulin for survival [1]. In contrast to insulin treatment, β-cell replacement through whole pancreas and islet transplantation is able to precisely maintain blood glucose levels in the physiological range [2]. Human islet transplantation has made remarkable progress and achieved high insulin independence rate at one year [3,4]. However, its long-term results revealed that only 15% of the recipients remained insulin independent at nine years after transplant [5]. Even though, C-peptide was detectable in 73% of them which indicated the existence of grafted islets [5]. To assess islet engraftment and recognize graft loss early on, an accurate and reproducible noninvasive islet imaging is needed [6,7].

Nanomaterials such as superparamagnetic iron oxide (SPIO) [8], Gd(III) [9] and iron oxides and Gd(III) [10] nanoparticles (NPs) have been used as contrast agents for magnetic resonance imaging (MRI). MRI has been applied for the detection of transplanted islets labeled by SPIO NPs coated with dextran, such as ferumoxides (Feridex^®^, Endorem^TM^) and ferucarbotran (Resovist^®^) in mice [11,12,13,14], rats [15,16,17,18,19,20,21,22], baboons [23] and humans [24,25]. However, due to lack of clear benefits, efficacy, specificity and clinical data, Feridex^®^ and Resovist^®^ were withdrawn in 2008 and 2009, respectively [26]. Thus, it is important to develop new MR contrast agents for islet imaging. Previously, we developed a method for in situ coating of SPIO NPs with chitosan in order to increase the content of magnetite [27] and demonstrated the chitosan-coated SPIO (CSPIO) NPs can be a T2 contrast agent for labeling islet grafts [28]. We later showed that CSPIO-labeled MIN6 β-cells [29], porcine neonatal pancreatic cell clusters [30], as well as adult mouse islet isografts [31,32] and allografts [32,33] can be safely and effectively imaged by MR for a long period of time. However, the MR images of transplanted islets labeled with SPIO NPs are nonspecific for β-cells because SPIO NPs can be taken up and localize in islet α-, β-, δ-, PP-cells and macrophages [12,18,32].

The glucagon-like peptide-1 (GLP-1) receptor is exclusively expressed on the surface of islet β-cells but not α-, δ- and PP-cells in mouse, rat and human pancreas [34]. Several studies showed that high uptake of radio-labeled GLP-1 analog exendin-4 (Ex4) in pancreas and insulinomas detected by positron emission tomography (PET)/single photon emission computed tomography (SPECT) [35,36,37,38,39]. Notably, the visualization of islets transplanted in a woman’s muscle using radiolabeled Ex4 concluded that human β-cells can be imaged in vivo with a tracer specific to the GLP-1 receptor [40]. Regarding β-cell specific MRI probes, Zhang et al. conjugated Ex4 to PEG-SPIO and demonstrated its properties of binding β-cells and in vivo imaging of implanted insulinoma in mice [41]. Wang et al. demonstrated the targeted probe MN-Ex10-Cy5.5 could be used for in vivo imaging of pancreatic β-cells in prediabetic and diabetic NOD mice [42]. Vinet et al. developed an Ex4-nanoparticle probe, Np647-ExCys1, for repeated MR imaging native pancreatic islets in mice after diphtheria toxin administration [43]. However, this strategy has not been applied in imaging transplanted β-cells.

Manganese ion is an attractive contrast agent for MRI. It not only shortens the T1 of water protons, but also has a T2 effect [44]. A new class of iron oxide NPs, magnetism-engineered iron oxide (MEIO) NPs, has been created with high and tunable nanomagnetism [45]. These MEIO NPs were doped with manganese (MnMEIO NPs) to provide further MR signal enhancement. We further developed a nanocarrier consisting of a MnMEIO core and a copolymer shell of silane and amine-functionalized poly(ethylene glycol) (PEG) [46,47]. The flexible PEG arms can mask positive charges generated by the non-conjugated reactive amine groups and reduce nonspecific binding of MnMEIO-silane-NH2-mPEG NPs to cells. Moreover, conjugation of the reactive amine groups on MnMEIO-silane-NH2-mPEG NPs with epidermal growth factor receptor (EGFR) antibody could specifically and effectively target mouse EGFR-expressing tumors. In this study, we conjugated MnMEIO NPs with Ex4 constructing a potential β-cell-specific MRI probe, and investigated the targeting properties of MnMEIO-Ex4 NPs to MIN6 β-cell line in vitro and transplanted islet β-cells in vivo.

## 2. Materials and Methods

### 2.1. Materials

Collagenase type XI, exendin-4 (Ex4), Histopaque^®^-1077, manganese (II) chloride (MnCl_2_·4H_2_O, 99%), iron (III) acetylacetonate (Fe(acac)_3_, 99.9%), methyl poly(ethylene glycol) (mPEG, M.W. = 2000), *N*-hydroxysuccinimide (NHS), ***N***-ethyl-*N*′-(3-dimethylaminopropyl) carbodiimide (EDC), oleic acid (90%), oleylamine (90%), osmium tetroxide (1%), propidium iodide (PI) and Prussian blue were purchased from Sigma–Aldrich (St. Louis, MO, USA). *N*-Boc-ethylenediamine (98%) and Acryloyl chloride (96%) were from Alfa Aesar (Ward Hill, MA, USA). Benzyl ether and (3-Aminopropyl) triethoxy silane (APTES, 98%) were from Fluka (Buchs, Switzerland). *N*-hydroxybenzotriazole (HOBt) and (Benzotriazol-1-yloxy) tripyrrolidinophosphonium hexafluorophosphate (PyBOP) were from NovaBiochem (Beeston, UK). RPMI-1640 medium and Dulbecco’s modified Eagle’s medium (DMEM) were from GIBCO BRL (Grand Island, NY, USA). Insulin radioimmunoassay (RIA) kit was from Millipore Corporation (Billerica, MA, USA). Polyethylene (PE-50) tubing was from Clay Adams (Parsippany, NJ, USA). Guinea pig anti-swine insulin antibody and rabbit anti-human glucagon antibody were from Dako (Carpinteria, CA, USA). Guinea pig anti-human insulin antibody (ab7842) was from Abcam (Cambridge, MA, USA).

### 2.2. Synthesis of MnMEIO and MnMEIO-Ex4 NPs

**Figure 1:**
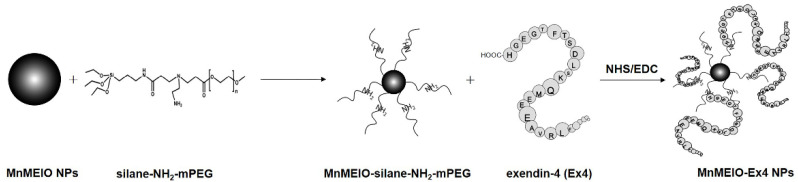
Schematic illustration of the synthetic process for MnMEIO-Ex4 NPs.

We have synthesized MnMEIO-silane-NH_2_-mPEG NPs [46,47]. To prepare functionalization MnMEIOs-Ex4 NPs, EDC (300 μL of 1.6 mg/mL) and NHS (300 μL of 1 mg/mL) were added to the Ex4 solution (20 μL of 1 mg/mL) containing MnMEIO-silane-NH_2_-mPEG NPs (Figure 1). The mixture was stirred by a mixer at 4 °C for 90 min and then the dialysis membrane (50 kDa) was used to remove the excess EDC, NHS and free antibody to obtain the MnMEIO-Ex4 NPs.

### 2.3. Characterization of MnMEIO and MnMEIO-Ex4 NPs

The hydrodynamic size and zeta potential were determined by dynamic light-scattering (DLS) with Zetasizer (Nano ZS90, Malvern Instruments, Malvern, UK). The iron concentration of ferrofluid was photometrically analyzed by o-phenanthroline and the absorbance was read at 510 nm [48]. Energy-dispersive spectra (EDS) of MnMEIO and MnMEIO-Ex4 NPs were measured by an energy-dispersive spectrometer (S-3000N, Hitachi, Tokyo, Japan).

### 2.4. Culture of MIN6 Cells

MIN6 cells, obtained from Prof. Susumu Seino at Kobe University, Kobe, Japan, were cultured in DMEM medium with supplementation of 10% fetal bovine serum (FBS). The cells were incubated at 37 °C in the presence of 5% carbon dioxide. The medium was changed every 3 days and cells were passaged weekly.

### 2.5. In Vitro Cytotoxicity Assay of MnMEIO and MnMEIO-Ex4 NPs

Cell death was determined by Propidium iodide (PI) which only enters cells with damaged membranes. For flowmetric analysis, MIN6 cells were stained with PI (50 µg/mL) and then analyzed by flow cytometry using a FACSCalibur (Becton Dickinson, Oxford, UK) equipped with a single argon ion laser emitting an excitation light at 488 nm wavelength. Data of 10,000 cells were obtained at a low flow rate and then analyzed by a software (CellQuest) [28,32].

### 2.6. Insulin Secretion of MIN6 Cells

MIN6 cells were incubated with MnMEIO or MnMEIO-Ex4 NPs (40 μg/mL) for 4 h. Then, the culture supernatant was measured with an insulin RIA kit.

### 2.7. Cellular Uptake of MnMEIO and MnMEIO-Ex4 NPs

MIN6 cells, 3T3 fibroblasts and RAW macrophages were incubated overnight with MnMEIO and MnMEIO-Ex4 NPs. After a washing process to remove the excess of NPs, the cells were stained with Prussian blue (5% potassium ferrocyanide and 5% HCl) and the labeling efficiency was examined using a microscope. Cells containing intracellular blue particles were considered labeled [28].

### 2.8. Transmission Electron Microscope (TEM) Measurements

The core size and size distribution of MnMEIO and MnMEIO-Ex4 NPs were measured by a TEM (JEOL JEM-2000 EX II, Tokyo, Japan) at a voltage of 100 kV. Aqueous solutions of NPs were drop-casted onto a 200-mesh copper grid and the grid was air-dried at room temperature before TEM measurements [46].

MIN6 cells treated with NPs were prepared as following for TEM analysis: 1 × 10^6^ cells grown on glass coverslips were incubated with MnMEIO or MnMEIO-Ex4 NPs (10 μg/mL) for 24 h. After being washed with PBS to remove unbounded NPs, cells were trypsinized, washed and fixed in 2.5% glutaraldehyde for 2 h. Fixed cells were stained with 1% osmium tetroxide at room temperature for 1 h. Then, they were dehydrated by alcohol and embedded in epoxy resin for sectioning. Ultra-thin sections were stained with uranyl acetate and lead citrate and then examined with an electron microscope (Hitachi H-7500) [28].

Elemental analysis was performed by electron energy loss spectroscopy (EELS) in a TEM (JEOL 2010F, Tokyo, Japan) equipped with a Schottkytype emission gun and a Gatan imaging filter (GIF Trim, Pleasanton, CA, USA) [32].

### 2.9. In Vitro MR Scanning

MR imaging were obtained with a 7.0 T MRI (Bruker, Ettlingen, Germany). 1 × 10^6^ MIN6 cells were incubated overnight with MnMEIO or MnMEIO-Ex4 NPs at 37 °C. Samples were scanned by using a fast gradient recalled echo pulse sequence (repetition time (TR)/echo time (TE) = 881 msec/9.37 msec). The contrast enhancement was calculated by the following equation: contrast enhancement (%) = (SIpost − SIpre)/SIpre × 100, where SIpost is the signal intensity measured from within the phantom of cells incubated with the contrast agents, MnMEIO and MnMEIO-Ex4 NPs, and SIpre is the signal intensity for cells alone [28,30].

### 2.10. Animals

Male inbred C57BL/6 mice were purchased from the National Laboratory Animal Center, Taipei, Taiwan. Mice aged 8–12 weeks were used as islet donors and recipients. The animal experiment was approved by the Institutional Animal Care and Use Committee of Chang Gung Memorial Hospital, Taoyuan, Taiwan (No. 2015062902).

### 2.11. Islet Isolation and Labeling

With the mouse under anesthesia with sodium amobarbital, each pancreas was injected with 1.5 mg/mL of collagenase, and then removed and incubated in a water bath at 37 °C. Islets were purified by a density gradient Histopaque^®^-1077, and then handpicked under a dissecting microscope [28,31,32,33].

Isolated islets were incubated overnight in the RPMI-1640 medium containing 30 or 40 μg/mL MnMEIO and MnMEIO-Ex4 NPs at 37 °C in a 5% CO_2_ atmosphere. After that, islets were washed with culture medium and then used for islet transplantation.

### 2.12. Islet Transplantation

We syngeneically transplanted islets labeled with MnMEIO-Ex4 NPs beneath the kidney capsule of three nondiabetic C57BL/6 mice, each with 300, 600 or 700 islets. Islets in PE-50 tubing were centrifuged. With the mouse under anesthesia, a lumbar incision was made to expose the left kidney. Capsule was cut in the lower pole, and the tip of PE-50 tubing was advanced beneath the capsule to the upper pole, the final injection site of islets. The capsule was left unsutured [28,31,32,33].

### 2.13. In Vivo MR Scanning

After transplantation, serial MR imaging were carried out on a 7.0 T MRI scanner in three recipients using a surface coil with the following parameters for the gradient-recalled echo sequence: slice thickness = 0.5 mm, TR = 3700 msec, TE = 37 msec. MR signal intensity of the graft at the left kidney and the mirror area at the right kidney, a within-subject control, was calculated [28,29,30,31,32,33].

### 2.14. Islet Graft Removal and Histological Studies

The six-hundred- and seven-hundred-islet grafts were removed at 49 and 120 days after transplant, respectively. With the mouse under anesthesia, an abdominal incision was made to expose the left kidney. Then, the graft was excised with the adherent capsule.

The removed graft was fixed in a formalin solution and then processed for paraffin embedding and sectioning. Sections were stained for endocrine α- and β-cells with glucagon and insulin antibodies and for iron with Prussian blue [29,30,31,32,33].

### 2.15. Statistical Analysis

In vivo MR signal intensity was expressed as the mean and standard deviation (SD). Statistical analyses were conducted with the software PASW Statistics 21 (IBM SPSS Statistics for Windows; Armonk, NY, USA: IBM Corp.). The normal distribution of the variable was checked with the Kolmogorov-Smirnov test. A pair comparison of mean values of the graft at the left kidney and the mirror area at the right kidney, the unpaired student’s t-test was carried out if both samples passed the normality test. The Mann-Whitney U test (Wilcoxon test) was used if any one sample of the comparison pair failed with the normality test. A *p*-value less than 0.05 was considered significant.

## 3. Results

### 3.1. General Characterization of MnMEIO and MnMEIO-Ex4 NPs

**Figure 2:**
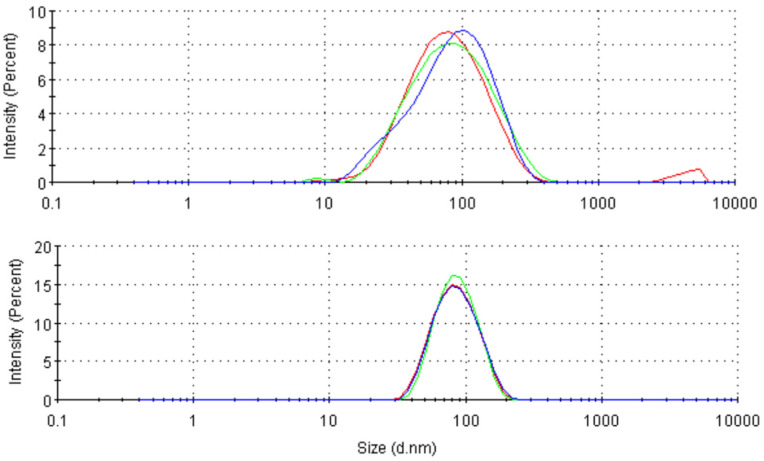
The hydrodynamic size distribution of MnMEIO NPs (**A**) and MnMEIO-Ex NPs (**B**). Three curves in black, green and red indicate three measurements.

We developed and characterized MnMEIO and MnMEIO-Ex4 NPs. The hydrodynamic size distribution, polydispersity index (PDI), zeta potential and iron concentration of MnMEIO were 67.8 ± 1.3 nm, 0.323 ± 0.04, 33.3 ± 0.5 mV and 0.91 mg/mL, respectively. Those of MnMEIO-Ex4 were 70.2 ± 2.3 nm, 0.36 ± 0.01, 0.6± 0.1 mV and 0.43 mg/mL, respectively (Table 1). Both of silane-EA-mPEG coated MnMEIO and MnMEIO-Ex4 NPs have a narrow size distribution (Figure 2).

### 3.2. Effects of MnMEIO and MnMEIO-Ex4 NPs on MIN6 Cell Viability and Insulin Secretion

**Figure 3:**
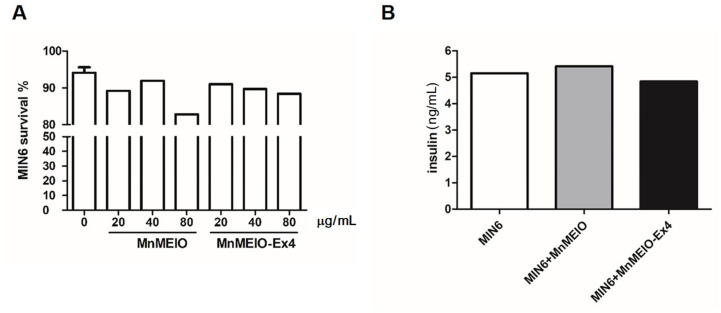
Effects of MnMEIO and MnMEIO-Ex4 NPs on MIN6 cell viability and insulin secretion. (**A**) Dose effects of MnMEIO and MnMEIO-Ex4 NPs on the viability of MIN6 cells. (**B**) Effects of 40 μg/mL MnMEIO and MnMEIO-Ex4 NPs on insulin secretion of MIN6 cells.

We utilized PI staining to analyze the toxicity of MnMEIO and MnMEIO-Ex4 NPs to MIN6 cells. Figure 3A shows MIN6 cell viability maintained in culture with 20 and 40 μg/mL of MnMEIO and MnMEIO-Ex4 NPs, however, some cytotoxicity appeared in MIN6 cells with 80 μg/mL of MnMEIO NPs. Based on this finding; we used MnMEIO and MnMEIO-Ex4 NPs ≤ 40 μg/mL for the rest of our in vitro and in vivo experiments.

Insulin secretion of MIN6 cells was measured 4 h after incubating MIN6 cells with MnMEIO and MnMEIO-Ex4 NPs (40 μg/mL). The insulin levels were not significantly different among MIN6 cells with or without MnMEIO and MnMEIO-Ex4 NPs (Figure 3B).

### 3.3. Cellular Uptake of MnMEIO and MnMEIO-Ex4 NPs

**Figure 4:**
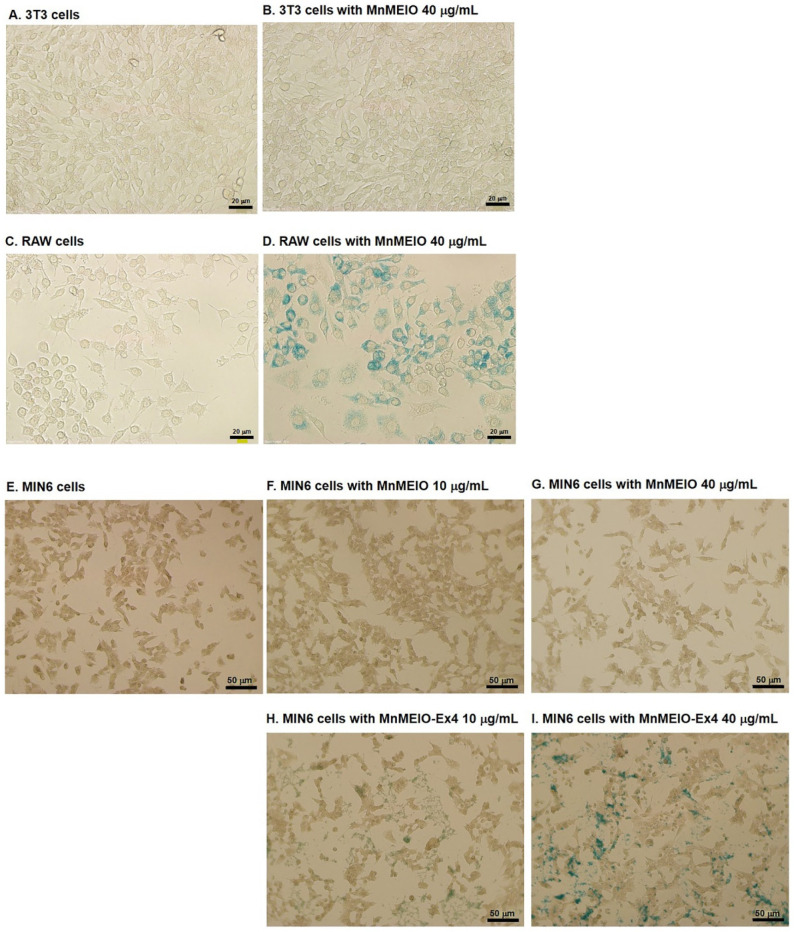
Cellular uptake of MnMEIO and MnMEIO-Ex4 NPs NPs. The 3T3 fibroblasts (**A**) and RAW macrophages (**C**) were incubated overnight with MnMEIO NPs 40 μg/mL (**B**,**D**). MIN6 cells (**E**) were incubated overnight with MnMEIO NPs 10 μg/mL (**F**), 40 μg/mL (**G**) and MnMEIO-Ex4 NPs 10 μg/mL (**H**), 40 μg/mL (**I**). The intracellular iron content was examined by Prussian blue staining.

**Figure 5:**
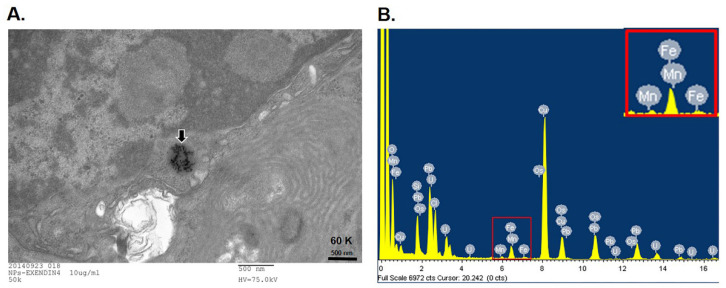
TEM micrographs of MIN6 cells incubated with 10 μg/mL MnMEIO-Ex4 NPs. (**A**) Electron dense particles in an endocytotic vesicle was indicated by the arrow. (**B**) Elemental analysis by electron energy-loss spectroscopy in MIN6 cells labeled with 10 μg/mL MnMEIO-Ex4 NPs. The peaks of manganese and iron were magnified in the inset box at top right corner.

We first examined the uptake of MnMEIO NPs by non-β cells, 3T3 fibroblasts (Figure 4A,B) and RAW macrophages (Figure 4C,D). After being incubated overnight with NPs, cells were stained with Prussian blue. There was no intracellular iron staining in 3T3 fibroblasts (Figure 2B) but it was positive in RAW macrophages (Figure 4D). We then examined the uptake of MnMEIO and MnMEIO-Ex4 by MIN6 β-cells (Figure 4E–I). There was no iron stain found in MIN6 cells with MnMEIO loading (Figure 4F,G). In contrast, the blue spots were observed in MnMEIO-Ex4 NPs-labeled MIN6 cells (Figure 4H,I), especially those with 40 μg/mL (Figure 4I). Our TEM results further confirmed electron dense particles in the cytoplasm of MnMEIO-Ex4 NPs-loaded MIN6 cells (Figure 5A). Moreover, elemental analysis by EELS demonstrated the presence of manganese and iron in MIN6 cells labeled with MnMEIO-Ex4 NPs (Figure 5B).

### 3.4. In Vitro MR Images of MnMEIO and MnMEIO-Ex4 NPs and NPs-Labeled MIN6 Cells

**Figure 6:**
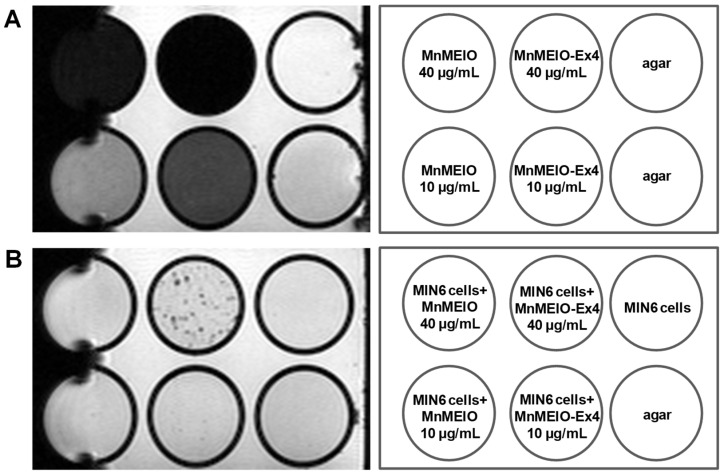
In vitro MR image of (**A**) 10 and 40 μg/mL MnMEIO and MnMEIO-Ex4 NPs and (**B**) their loading MIN6 cells. All were scanned by a 7.0 T MRI system. Agar was used as a negative control and MnMEIO and MnMEIO-Ex4 NPs were used as a positive control. In contrast to MnMEIO NPs, MnMEIO-Ex4 NPs-loaded MIN6 cells appeared as dark spots.

We performed in vitro MRI on agar, MnMEIO and MnMEIO-Ex4 NPs (Figure 6A) as well as MIN6 cells incubated with or without MnMEIO and MnMEIO-Ex4 NPs (Figure 6B). As expected, there was a background image of the agar (a negative control) and a dark image of MnMEIO and MnMEIO-Ex4 NPs (a positive control) (Figure 6A). In contrast to a background image in MIN6 cells and MnMEIO NPs-labeled MIN6 cells, MnMEIO-Ex4 NPs-labeled MIN6 cells appeared as dark spots, especially those with 40 μg/mL (Figure 6B).

### 3.5. In Vivo MR Images of MnMEIO-Ex4 NPs-Labeled Islets after Transplantation

**Figure 7:**
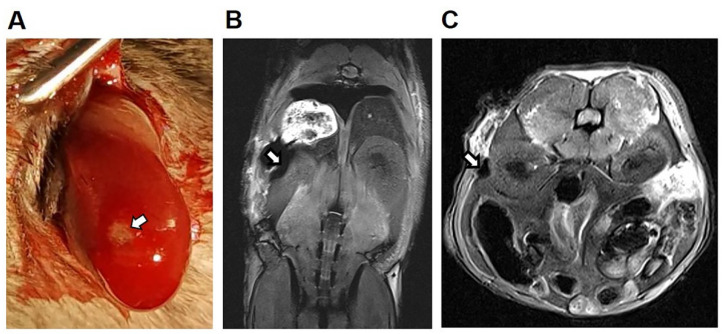
In vivo MR image of the MnMEIO-Ex4 NPs-labeled islet graft on the second day of transplantation. (**A**) Three hundred MnMEIO-Ex4 NPs-labeled islets were transplanted under the left kidney capsule of a C57BL/6 mouse. The recipient was scanned by a 7.0 T MRI system with coronal (**B**) and transverse (**C**) sections on the second day of transplantation. The graft was indicated by arrows.

**Figure 8:**
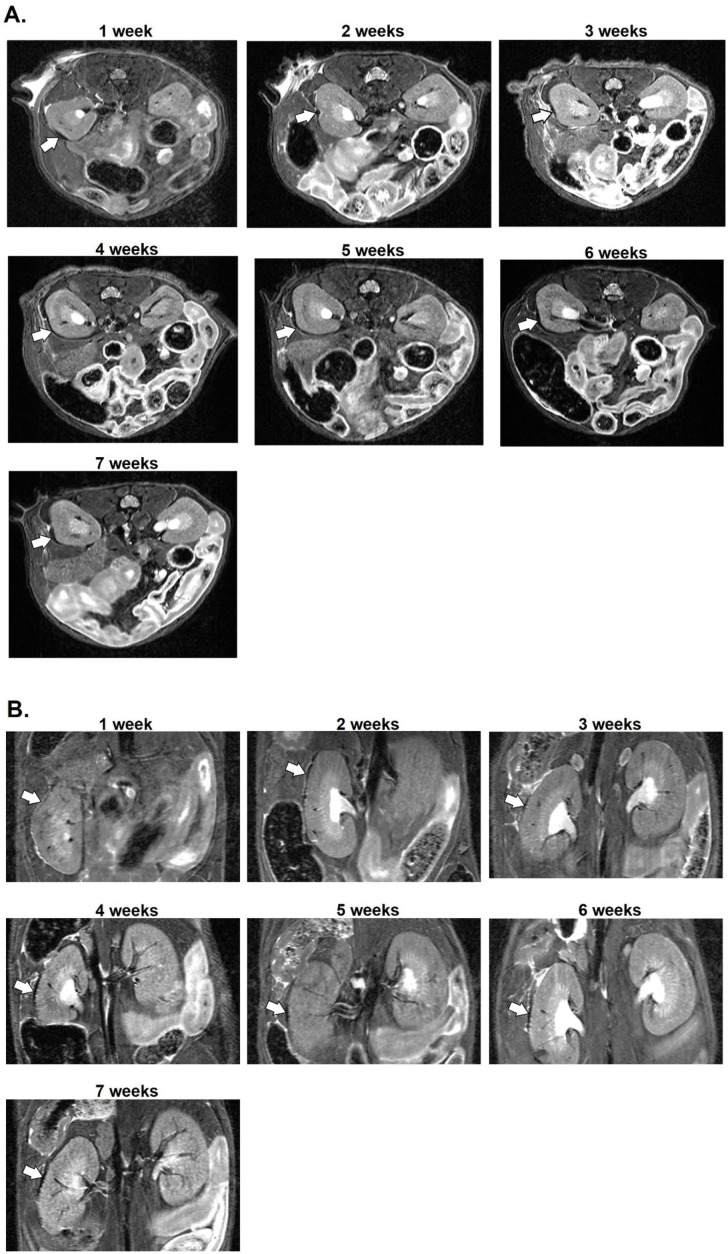

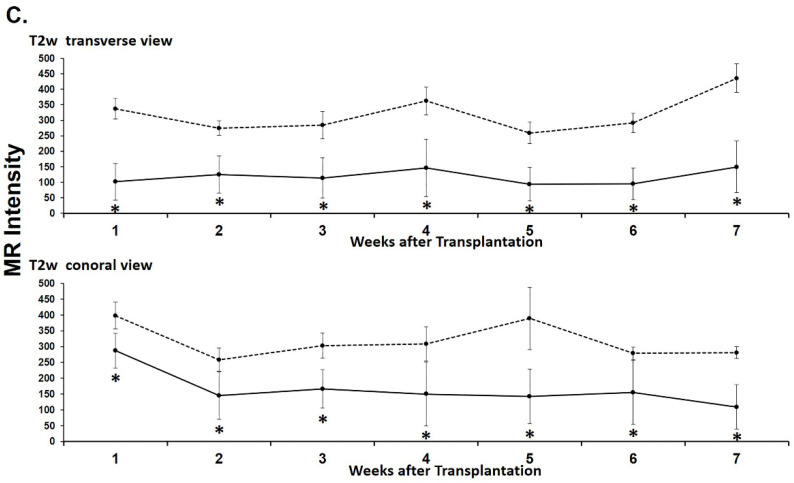
Seven-week in vivo MR images of the MnMEIO-Ex4 NPs-labeled islet graft. Six hundred MnMEIO-Ex4 NPs-labeled islets were transplanted under the left kidney capsule of a C57BL/6 mouse. The recipient was scanned by a 7.0 T MRI system with transverse (**A**) and coronal (**B**) sections for 7 weeks. The graft on MR images was indicated by arrows. (**C**) Time course of the MR signal intensity of the graft on left kidney (solid line) and the mirror area on the right kidney (dash line) in the mouse transplanted with 600 MnMEIO-Ex4 NPs-labeled islets. * *p* = 0.000.

**Figure 9:**
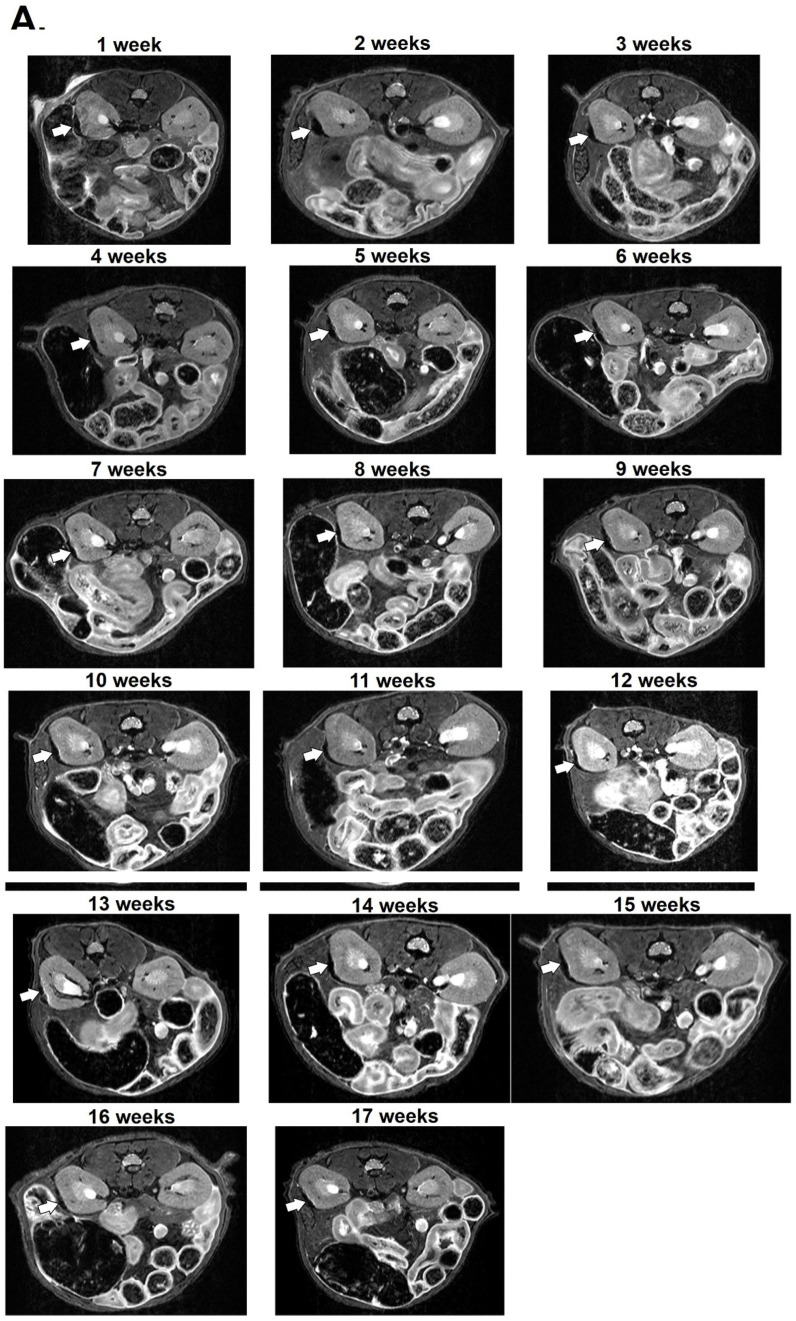

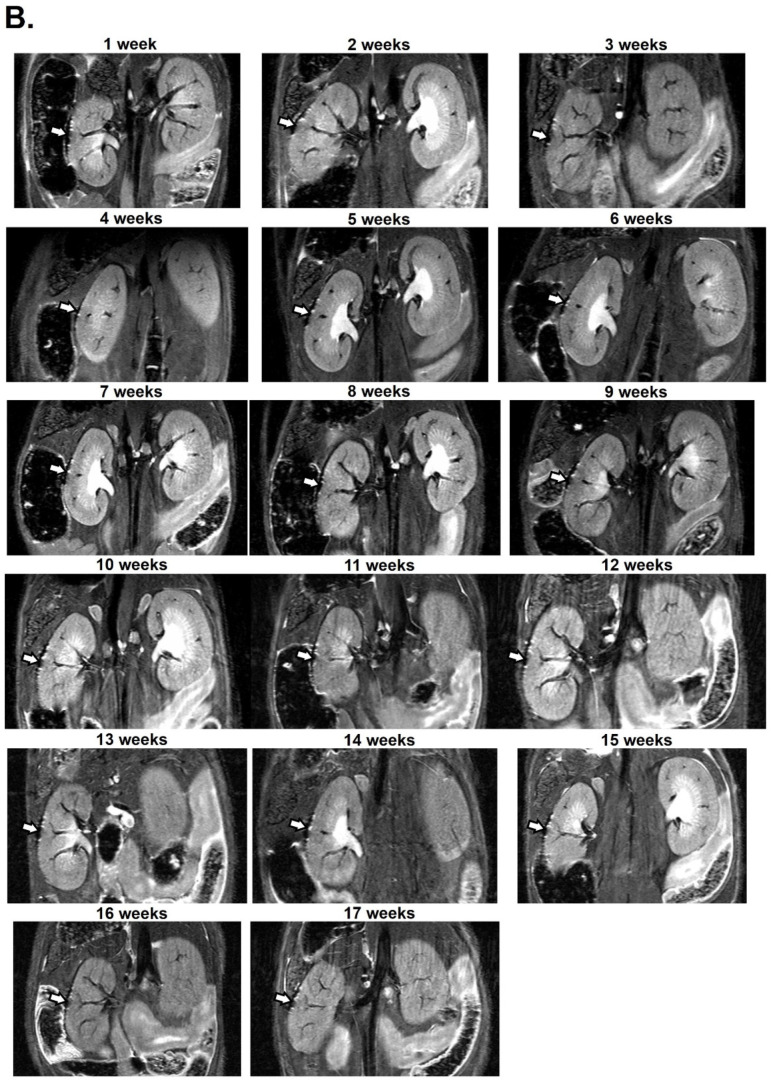

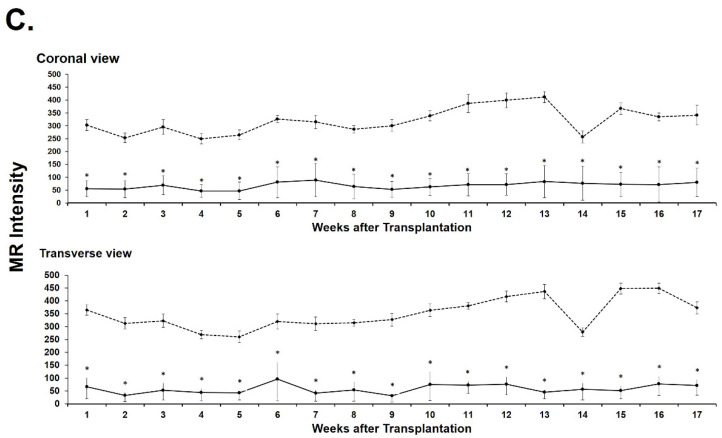
Seventeen-week in vivo MR images of the MnMEIO-Ex4 NPs-labeled islet graft. Seven hundred MnMEIO-Ex4 NPs-labeled islets were transplanted under the left kidney capsule of a C57BL/6 mouse. The recipient was scanned by a 7.0 T MRI system with transverse (**A**) and coronal (**B**) sections for 17 weeks. The graft on MR images was indicated by arrows. (**C**) Time course of the MR signal intensity of the graft on left kidney (solid line) and the mirror area on the right kidney (dash line) in the mouse transplanted with 600 MnMEIO-Ex4 NPs-labeled islets. * *p* = 0.000.

Since MIN6 cells did not uptake MnMEIO NPs in our in vitro study, islets labeled with MnMEIO-Ex4 NPs were used for syngeneic transplantation in three nondiabetic C57BL/6 mice. In our pilot study, we transplanted 300 islets labeled with MnMEIO-Ex4 NPs under the left kidney capsule of one mouse and performed MRI on the second day (Figure 7). On MR scans, the graft of MnMEIO-Ex4 NPs-labeled islets (indicated by arrows) was visualized as a distinct hypointense area located at the implantation site. Then, we performed long-term studies by transplanting 600 and 700 islets labeled with MnMEIO-Ex4 NPs into each of two mice and followed-up MRI between 1 and 7 weeks in the former (Figure 8) and between 1 and 17 weeks in the latter (Figure 9). In both mice, the above-mentioned hypointense areas were persistent on MR scans. Our quantification analysis further revealed that, in two mice, the MR signal intensity of the MnMEIO-Ex4 NPs-labeled islet grafts made a persistent 30–70% (Figure 8C) and 70–90% reduction (Figure 9C) of MR signal as compared to the same area in the contralateral kidney at all time points (*p* = 0.000). This indicates the potential for long-term monitoring of islet isografts with the use of MnMEIO-Ex4 NPs.

### 3.6. Histological Studies of the MnMEIO-Ex4 NPs-Labeled Islet Graft

**Figure 10:**
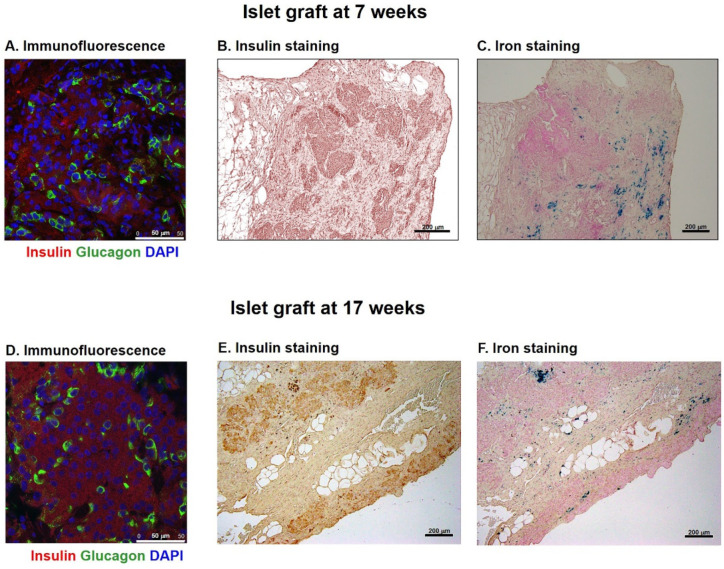
Histology of the MnMEIO-Ex4 NP-labeled islet graft removed at 7 (**A**–**C**) and 17 weeks (**D**–**F**) after transplantation. (**A**,**D**) Immunofluorescence staining of insulin (red color) and glucagon (green color) in islet grafts. (**B**,**E**) Grafts were stained with insulin (brown color). (**C**,**F**) Grafts were stained with Prussian blue (blue color).

MnMEIO-Ex4 NPs-labeled islet grafts were removed from two recipients at 7 and 17 weeks after transplantation, respectively. To investigate the graft microscopically, we used a glucagon and an insulin antibody to stain islet α- and β-cells, respectively, and Prussian blue to stain iron. There were abundant insulin- and glucagon-positive cells in islet grafts (Figure 10A,B,D,E). Moreover, these grafts were also stained positive for iron (Figure 10C,F).

## 4. Discussion

The β-cell-specific imaging is important for understanding the fate of β-cells after islet transplantation. In this study, we conjugated MnMEIO NPs with Ex4 constructing a potential β-cell-specific MRI probe, MnMEIO-Ex4 NPs, and demonstrated its targeting properties to MIN6 β-cell line in vitro and transplanted islet β-cells in vivo. An ideal cell tracking agent should not associate with incidental adverse effects. Previously, Lee et al. reported MnMEIO NPs were biologically nontoxic to HeLa and HepG2 cell lines [41]. In the present study, we further demonstrated the concentration of MnMEIO and MnMEIO-Ex4 NPs ≤ 40 μg/mL did not affect MIN6 cell viability and insulin secretion, which is essential for in vivo islet transplantation.

The size of MnMEIO and MnMEIO-Ex4 NPs is around 70 nm which is between two previously commercial SPIO NPs, ferumoxides (120–180 nm) and ferucarbotran (30 nm) [26]. SPIO NPs are efficiently internalized by different cell types through uptake routes including pinocytosis, phagocytosis and receptor-mediated endocytosis [26]. In non-β cells, RAW macrophages instead of 3T3 fibroblasts could uptake MnMEIO NPs, implying MnMEIO NPs enter cells via phagocytosis but not by pinocytosis. In MIN6 β-cells, MnMEIO-Ex4 but not MnMEIO NPs were taken up, indicating MIN6 cells uptake MnMEIO NPs through receptor-mediated endocytosis. These densely packed MnMEIO-Ex4 NPs in MIN6 cells were confirmed by the TEM and elemental analysis by EELS. They were responsible for producing a local magnetic field and resulted in higher contrast in MR images as dark spots, which were reported corresponding to the locations of single loaded cells [28]. This visualization of MnMEIO-Ex4 NPs-labeled MIN6 cells is fundamental for detecting islet grafts by in vivo MRI.

**Figure 11:**
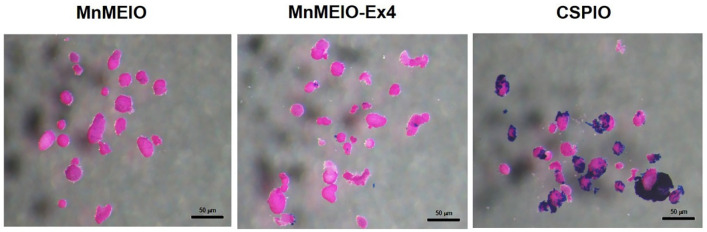
Binding of MnMEIO, MnMEIO-Ex4 and CSPIO NPs to islets. Islets were incubated overnight with MnMEIO NPs 40 μg/mL, MnMEIO-Ex4 NPs 40 μg/mL and CSPIO 10 μg/mL, respectively, and then stained with Prussian blue.

In our in vivo study, we syngeneic transplanted islets labeled with MnMEIO-Ex4 NPs under the left kidney capsule of three nondiabetic C57BL/6 mice. On MR scans, all MnMEIO-Ex4 NPs-labeled islet grafts were visualized as a distinct hypointense area located at the implantation site. Even though, we found that the hypointense area was bigger on the second day than those at 1 week and later after transplantation, that may be due to islet loss caused by death from anoxia and the lack of engraftment soon after implantation [49]. The persistent hypointense areas on the MR images during 7- and 17-week follow-ups are consistent with our 18-week observation in CSPIO-labeled islet isografts [28,29]. However, MnMEIO-Ex4-labeled islet grafts had smaller hypointense areas on MR images. This could be explained firstly by the strong binding of cationic chitosan with anionic cell surface which enhanced internalization of CSPIO NPs via endocytosis [50]. As shown in Figure 11, there were more CSPIO NPs adhered to islet surface than MnMEIO and MnMEIO-Ex4 NPs did. Secondly, the uptake of MnMEIO-Ex4 NPs is β-cell specific but CSPIO NPs can be taken up by any cells in islets. Therefore, there were fewer MnMEIO-Ex4 NPs-containing cells than CSPIO NPs-containing cells in the islet graft. Our quantification analysis in two mice further confirmed a persistent reduction of the MR signal intensity of the MnMEIO-Ex4 NPs-labeled islet grafts as compared to the same area in the contralateral kidney during 7- and 17-week follow-ups, respectively. This indicates the potential for long-term monitoring of islet isografts with the use of MnMEIO-Ex4 NPs. Although the Ex4-NP probes were used for in vivo MR imaging implanted insulinoma [41] and native pancreatic β-cells [42,43], to the best of our knowledge, we are the first to apply this strategy by using MnMEIO-Ex4 NPs to image islet grafts for a long period of time.

The histological studies of MnMEIO-Ex4 NPs-labeled islet grafts revealed abundant insulin- and glucagon-positive cells in islet grafts, indicating the existence of functioning islets at 7 and 17 weeks after transplantation, respectively. In addition, positive iron staining implies that MnMEIO-Ex4 NPs in transplanted islets were responsible for positive MR images. Taken together, MnMEIO-Ex4 NPs are potential for the in vivo molecular targeting of transplanted β-cells.

## 5. Conclusions

To detect and trace transplanted β-cells by MRI, we conjugated MnMEIO NPs with exendin-4 which can specifically bind the GLP-1 receptors on β-cells. MnMEIO-Ex4 NPs did not affect MIN6 cell viability and insulin secretion and could be specifically taken up by MIN6 cells. Both MnMEIO-Ex4-labeled MIN6 cells and islet grafts showed positive MR images in vitro and after transplantation, respectively. Moreover, histology of the islet graft showed positive staining for insulin, glucagon and iron. Our results indicate that MnMEIO-Ex4 NPs are safe and effective for the detection and long-term monitoring of transplanted β-cells by MRI.

## Figures and Tables

**Table 1:** Zeta potential and hydrodynamic size of nanoparticles.

Materials	Hydrodynamic Size (nm)	Zeta Potential (mV)
MnMEIO NPs	24.2 ± 2.3	−5.1 ± 0.3
MnMEIO (MnMEIO-silane-NH_2_-mPEG)	67.8 ± 1.3	33.3 ± 0.5
MnMEIO-Ex4 (MnMEIO-silane-NH_2_-mPEG-Ex4)	70.2 ± 2.3	0.6 ± 0.1

## Data Availability

Not applicable.
